# Particle Image Velocimetry (PIV) Investigation of the Turbulent Airflow in Slot-Die Melt Blowing

**DOI:** 10.3390/polym12020279

**Published:** 2020-01-31

**Authors:** Sheng Xie, Guojun Jiang, Baolin Ye, Baoqing Shentu

**Affiliations:** 1 College of Material and Textile Engineering, Jiaxing University, Jiaxing 314001, China; jiangguojun1986@126.com; 2 State Key Lab of Chemical Engineering, College of Chemical and Biological Engineering, Zhejiang University, Hangzhou 310027, China; 3 College of Mechanical and Electrical Engineering, Jiaxing University, Jiaxing 314001, China; yebaolin@zjxu.edu.cn

**Keywords:** turbulent airflow, melt blowing, PIV, microfiber, whipping motion

## Abstract

In order to explore the forming mechanism of the fiber whipping motion in slot-die melt blowing, the turbulent airflow in slot-die melt blowing was measured online with the approach of the Particle Image Velocimetry (PIV) technique. The PIV results visualized the structure of the turbulent airflow and provided the distributions of air velocity components (*v*_x_, *v*_y_, and *v*_z_). Moreover, the PIV results also demonstrated the evolutive process of turbulent airflow at successive time instants. By comparing the characteristics of the turbulent airflow with the fiber whipping path, the PIV results provide a preliminary explanation for the specific fiber whipping motion in slot-die melt blowing.

## 1. Introduction

Melt-blown microfibers with a mean diameter of several micrometers are obtained by attenuating the polymeric melt jets with the help of a high-speed air stream [1], and the melt-blown micro-fibrous materials are widely used in areas of filtration and oil/water separation [2,3]. Recently, functional fibers, such as melt-blown cross-linked or helical fibers, have emerged [4,5], which are expected to have great potential applications.

The principle of the melt blowing reveals that the research on the melt-blown airflow is significant, and many researchers have done a lot of work in this field with different approaches. The initial measurement of melt-blown airflow was performed using a pitot tube [6,7,8,9,10,11]. The pitot tube measurements showed the velocity distribution of the low velocity flow field. The second generation of equipment measuring the melt-blown airflow are the laser doppler velocimeter and hot-wire anemometer [12,13]. These two pieces of equipment could measure the airflow with a higher air velocity. Note that the hot-wire anemometer was also used for measuring the melt-blown fluctuating air velocity [14] and the fluctuating air temperature [15]. In addition, the diffusion angle of the airflow in slot-die melt blowing was tentatively measured using a dual-probe hot-wire anemometer, and the results showed that the lateral velocity component was significant for initiating fiber whipping [16]. It is noted that all this research on airflow belongs to asynchronous measurement, which was carried out by measuring the airflow at discrete points one by one. The spatial distribution of velocity at a time instant is still unavailable, resulting in the exploration of melt-blown airflow being still incomplete until now.

In addition to the experimental measurements, Computational Fluid Dynamics (CFD) was another productive approach for investigating the melt-blown airflow. The CFD simulations of melt-blown airflow were firstly carried out in 2002. At that time, Shambaugh and Papavassiliou et al. [17] simulated the time-averaged melt-blown airflow under isothermal conditions. In the same year of 2002, Mukhopadhyay et al. [18] simulated the turbulent airflow in sharp-die melt blowing with a large eddy simulation (LES) approach. Their work firstly demonstrated the turbulent airflow in melt blowing. It is regretful that the subsequent CFD studies [19,20,21,22,23,24] were concentrated on simulating the time-averaged flow field, rather than the dynamic turbulence, indicating that the CFD simulations on air turbulence is very inadequate, and there is still a lot of CFD work to be done on melt-blown turbulent airflow.

The importance and urgency of air-turbulence research can be reflected by the discovery of the fiber whipping motion during the melt-blown process. Although the studies on the fiber whipping motion are less than those on the flow field, discoveries on fiber whipping can be briefly summarized as follows: (1) the fiber path during the slot-die melt blowing is composed of alternating left–right arcs [25]; (2) the fiber path laterally expands as it moves to the collector [16,26]; and (3) the lateral expansion of the fiber path is anisotropic [16]. More detailed descriptions of melt-blown fiber whipping also can be found in the reviews of Hao [27] and Drabek [28]. However, the special fiber whipping motion in slot-die melt blowing has not been thoroughly explained so far. Some theoretical works revealed that the research on turbulent airflow was the fundamental approach to solve the fiber whipping motion or fiber attenuation [29,30].

The present work focused on experimental exploring of the turbulent airflow in slot-die melt blowing, and the ultimate goal of this work is to explain the discovered fiber whipping motion [25]. The turbulent airflow was measured with the approach of Particle Image Velocimetry (PIV). The PIV technique has advantages of visualizing the turbulent airflow and measuring the spatial distribution of velocity at a time instant, which are difficult to accomplish by pitot tube, hot-wire anemometer, and laser doppler velocimeter techniques [11,12,13]. Although the PIV technique was widely used in the field of fluid mechanics [31,32], to the best of our knowledge, the PIV technique was firstly applied on measuring the melt-blowing airflow. In this work, the PIV results visualized the structure of the turbulent airflow and provided the distributions of air velocity components (*v*_x_, *v*_y_, and *v*_z_) in slot-die melt blowing. In addition, the PIV results showed the evolutive process of turbulent airflow at successive time instants. Finally, the specific fiber whipping motion in slot-die melt blowing was preliminarily explained by comparing the structures of the turbulent airflow with the fiber whipping path.

## 2. Experiments and Measurements

### 2.1. Melt-Blown Spinning Device

**Figure 1:**
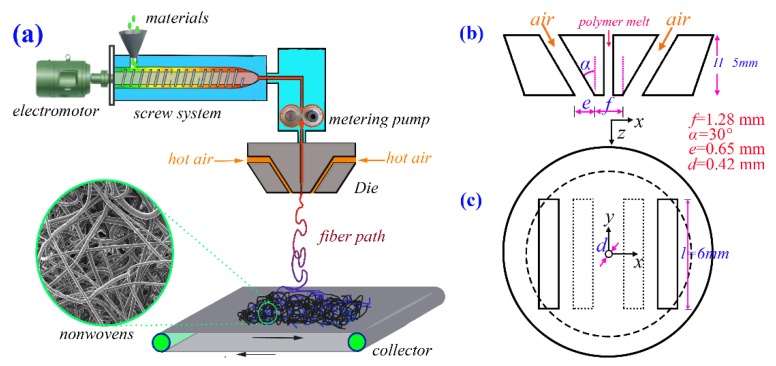
(**a**) Schematic process of the slot-die melt blowing. (**b**) Section view and (**c**) top view of the slot die used in the present work. The dot-lines in (**c**) are on the die face. This slot die is called “spinning die” in this work.

As shown in Figure 1a, a single-orifice melt-blown spinning device was used for the research of the fiber whipping motion in the previous works [25]. During the spinning process, the solid materials were firstly melted and extruded out through the polymer-orifice by an extruder; then, high-speed hot air streams attenuate the extruded polymeric melt into microfiber, which was deposited onto a collector in the form of nonwovens, as shown in Figure 1a. The type of extruder arranged in this device is a screw extruder. This type of extruder can ensure that: (1) the raw materials are evenly mixed; (2) there is no long time of melt retention, and the polymeric macromolecular cleavage can be avoided; and (3) the raw materials can be continuously added into the screw extruder, resulting in the continuity of the spinning process. In this device, the screw had an out-diameter of 12 mm and had the length–diameter ratio (L/D) of 36/1. Note that the previous work [25] focused on capturing the fiber whipping path via high-speed photography, rather than exploring the materials. Therefore, a melt flow rate of 650 g/10 min polypropylene (SK, Seoul, Korea) without additives was used as received for melt-blown spinning. The experimental conditions for the melt-blown spinning were described in detail in our previous work [25].

Figure 1b,c shows the section view and the top view of the slot die used for the research of the fiber whipping motion, and the structure parameters of this die are also added in Figure 1b,c. Note that the description of the same die also can be found in our previous works [16,25]. In order to distinguish this die from the amplified die, which will be described below, this die is called the “spinning die”.

### 2.2. PIV Measurement

**Figure 2:**
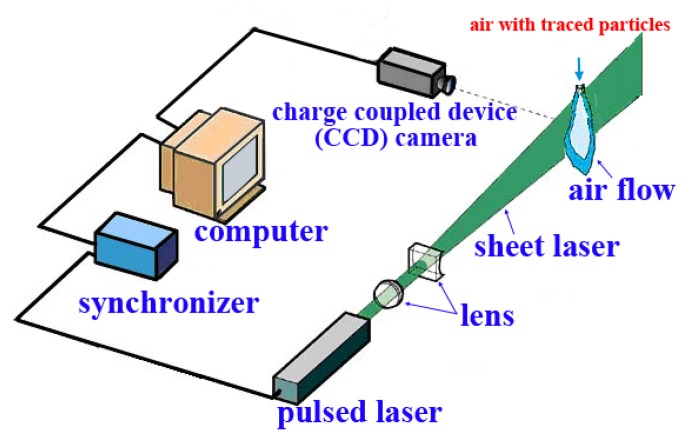
Schematic of the Particle Image Velocimetry (PIV) measurement system.

The melt-blown turbulent airflow was measured with the approach of Particle Image Velocimetry (i.e., PIV, from ILA Inc., Dallas, TX, USA). During the PIV measurement, as shown in Figure 2, the particle seeder emitted numerous traced particles (titanium dioxide, white, with a diameter of 0.3 µm) into the airflow. The pulsed laser emitted a pulsed laser, and with the help of refraction of the particular lens, the emitted laser became a sheet laser (i.e., laser in a plane). At the same time, the charge coupled device camera (i.e., CCD camera) captured the image of the airflow in the sheet laser. In this process, the synchronizer ensured that the time of CCD imaging was synchronized with the time of laser emission. After a very short time interval (Δ*t* = 60 µs), the pulsed laser emitted the next pulsed laser, and correspondingly, the airflow image was captured by the CCD camera again. The locations of the traced particles in the two images (“coupled images”) were recorded and analyzed; the displacements of the traced particles (Δ*r*) in Δ*t* were calculated. The instantaneous air velocity field in the plane of the sheet laser could be obtained by Δ*r*/Δ*t*. During the PIV measurement, the gauge pressure of the supplied air was 0.4 MPa, and the pressure meter was placed between the air compressor and the traced particle seeder. It is noted that the final purpose of this PIV measurement was to explore some relationships between the turbulent airflow and fiber whipping motion, therefore, the effect of air temperature on the turbulent airflow was not of concern this time (i.e., the PIV measurement was carried out in the condition of room temperature).

As shown in Figure 1b,c, the sectional size of the air orifice in the “spinning die” is less than 1 mm, which is too small to observe the macroscopic phenomena easily. For this PIV measurement, a magnified die (i.e., the “model die”) was made and used, which is five times larger than the “spinning die”, as shown in Figure 1b,c. Therefore, the amplified “model die” had a nose-piece width of 6.4 mm (*f* × 5), a slot width of 3.25 mm (*e* × 5), slot height of 25 mm (*H* × 5), and the slot length of 30 mm (*l* × 5). However, the slot angle was still the same, i.e., α = 30°, and there was no polymer orifice arranged in the “model die”. The PIV measurement using amplified “model die” was in accordance with the Reynolds similarity criterion, which can be described as

(1) $$\frac{\rho_{m}v_{m}L_{m}}{\mu_{m}}=\frac{\rho_{s}v_{s}L_{s}}{\mu_{s}}$$

where *ρ* is the air density, *v* is the air velocity, *μ* is the dynamic viscosity coefficient, and *L* is the characteristic scale or equivalent hydraulic diameter of the slot cross-section. The subscripts “m” and “s” are for the “model die” and the “spinning die”, respectively. Note that *ρ*_m_ = *ρ*, *μ*_m_ = *μ*, and *L*_m_ = 5*L*_s_, the airflow with air velocity of *v*_m_ for the “model-die”, can represent similar airflow with air velocity of 5 × *v*_m_ for the “spinning-die”.

**Figure 3:**
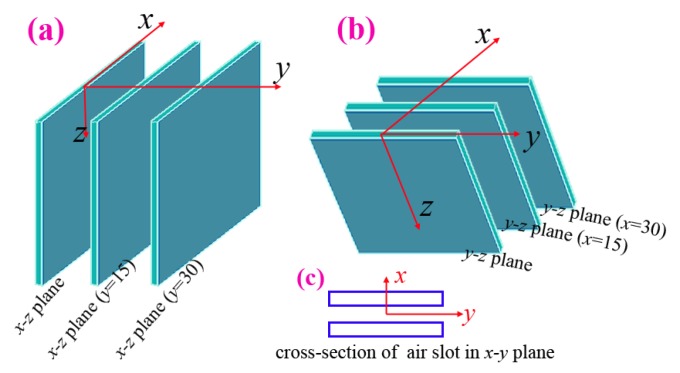
(**a**) Locations of three planes which are normal to the slot. They are the *x-z* plane, the “*x-z* plane (*y* = 15)”, and the “*x-z* plane (*y* = 30)”. (**b**) Another three planes which are parallel to the slot, i.e., the *y-z* plane, the “*y-z* plane (*x* = 15)”, and the “*y-z* plane (*x* = 30)”. (**c**) The cross section of air slots in the *x-y* plane.

In order to show the three-dimensional melt-blown air flow field, air flows in six spatial planes were measured. Figure 3a shows the first three planes which are normal to the slot and they are: (1) the *x-z* plane, (2) the plane which is parallel to the *x-z* plane and passes through the location of *y* = 15 mm, and (3) the plane which is parallel to the *x-z* plane and passes through the location of *y* = 30 mm. Here, the (2) and (3) planes are defined as “*x-z* plane (*y* = 15)” and “*x-z* plane (*y* = 30)” for short, respectively. Figure 3b shows another three planes which are parallel to the slot, namely, (4) the *y-z* plane, (5) the “*y-z* plane (*x* = 15)”, and (6) the “*y-z* plane (*x* = 30)”. In order to better understand the position relationship of the six planes, the cross-section of the air-slots is added as in Figure 3c.

## 3. Results and Discussion

### 3.1. Basic Structure of Turbulent Airflow

**Figure 4:**
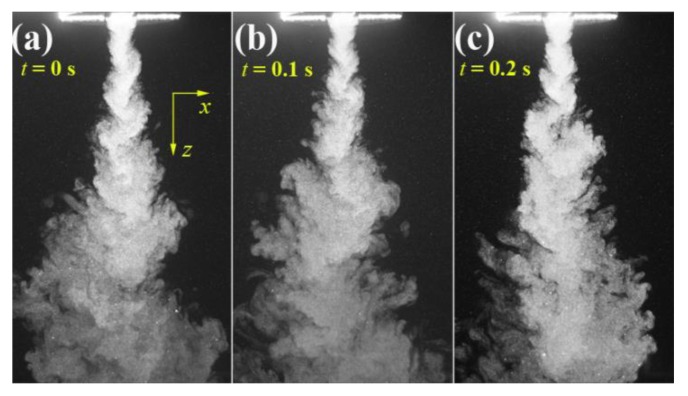
The visualized airflows in the *x-z* plane at different time instants of (**a**) *t* = 0 s, (**b**) *t* = 0.1 s, and (**c**) *t* = 0.2 s. The real size for each sub-image is 141.8 mm × 83.5 mm.

**Figure 5:**
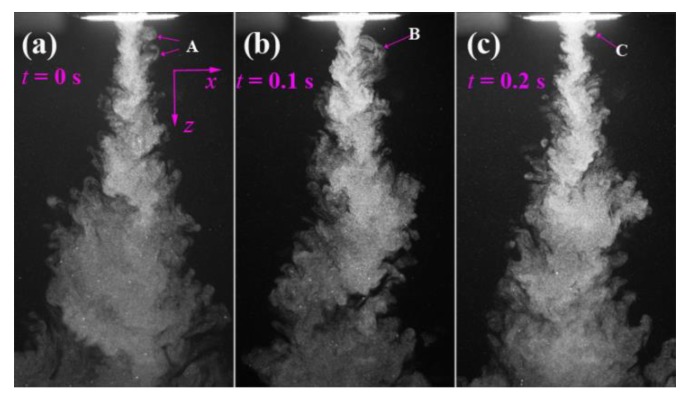
The visualized airflows in the “*x-z* plane (*y* = 15)” at different time instants of (**a**) *t* = 0 s, (**b**) *t* = 0.1 s, and (**c**) *t* = 0.2 s. The real size for each sub-image is 141.8 mm × 82.3 mm.

**Figure 6:**
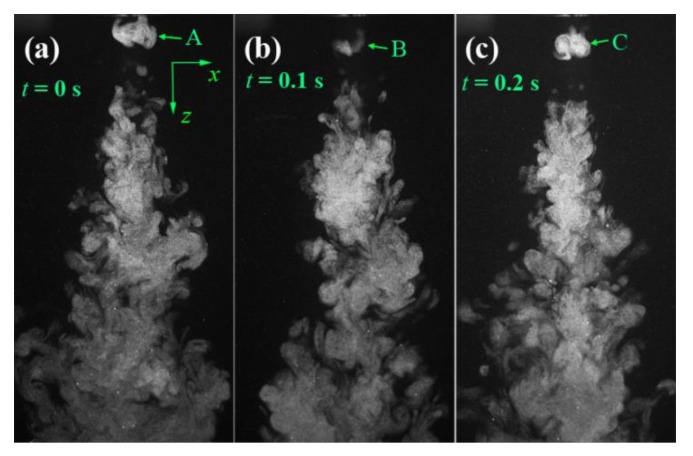
The visualized airflows in the “*x-z* plane (*y* = 30)” at different time instants of (**a**) *t* = 0 s, (**b**) *t* = 0.1 s, and (**c**) *t* = 0.2 s. The real size for each sub-image is 141.8 mm × 72.6 mm.

Figure 4 shows the visualized airflow in the *x-z* plane at time instants of *t* = 0, 0.1, and 0.2 s. The flow visualization shows that the ejected two air streams from a pair of slots integrate immediately and begin to form an S-shaped turbulent structure. However, further away from the die face, the S-shaped turbulent structure gradually weakens and evolves into disordered larger eddies, which are due to the accumulated air resistance of ambient air to the airflow. Figure 5 shows the visualized airflow in the “*x-z* plane (*y* = 15)” at time instants of *t* = 0, 0.1, and 0.2 s. Note the “*x-z* plane (*y* = 15)” passes through the edge of the air slot (the length of the slot is 30 mm, and the edges of the slot locate at *y* = ±15 mm). The integrated air stream also demonstrates an S-shaped turbulent structure in the region below the die face. However, the S-shaped turbulent structure is attached by a lot of small-scale eddies. These eddies are probably due to the airflow that was ejected from the edge of the slot having lower kinetic energy than the airflow ejected from the center of the slot, resulting in ambient air that can crash the S-shaped structure into small-scale eddies. Figure 6 shows the visualized airflow in the “*x-z* plane (*y* = 30)” at time instants of *t* = 0, 0.1, and 0.2 s. In this plane, the S-shaped structure of air turbulence disappears. The existence of the empty area below the die face in the visualized images, as shown in Figure 6, is due to that part of the airflow not existing in the “*x-z* plane (*y* = 30)”. Even so, a single eddy exists very near the die face, as “A”, “B”, and “C” in Figure 6 shows; this phenomenon indicates that there exists a lateral velocity component with a direction along the nose-piece or *y*-direction below the die face.

It is worth noting that the captured fiber whipping path in the *x-z* plane appears to consist of two groups of arcs (like a semi-ring) [25], which has a similar structure of turbulent airflow in the *x-z* plane, as shown in Figure 4. Therefore, the relationships between fiber whipping and turbulent airflow seems like that when the polymeric fiber comes out from the orifice, the fiber undergoes the force of the S-shaped airflow in the *x-z* plane and correspondingly begins to have the S-shaped whipping motion in the *x-z* plane.

**Figure 7:**
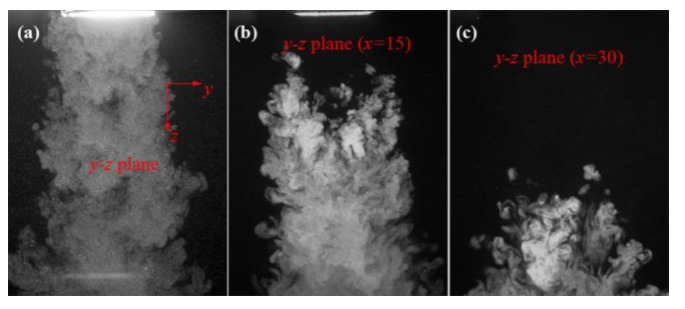
The visualized airflows in (**a**) the *y-z* plane, (**b**) the “*y-z* plane (*x* = 15)”, and (**c**) the “*y-z* plane (*x* = 30)”. The real size for each sub-image is 141.8 mm × 109 mm.

Figure 7 shows the visualized airflows in the *y-z* plane, the “*y-z* plane (*x* = 15)”, and the “*y-z* plane (*x* = 30)”. Figure 7a shows that the turbulent structure in the *y-z* plane is different to the turbulent structure in the *x-z* plane, as shown in Figure 4; the airflow in the *y-z* plane has another structure with numerous small-scale eddies on the airflow body, and the S-shaped eddy structure does not emerge. It worth noting that the airflow has a width larger than the diameter of the “model die” (the diameter of the “model die” face is 45 mm), which means the airflow has a lateral motion along the ± *y*-direction when the air is just coming out of the air-slot. Figure 7b,c shows that more small-scaled eddies emerge in the region laterally far away from the central *y-z* plane.

### 3.2. Velocity Distribution of Turbulent Airflow

**Figure 8:**
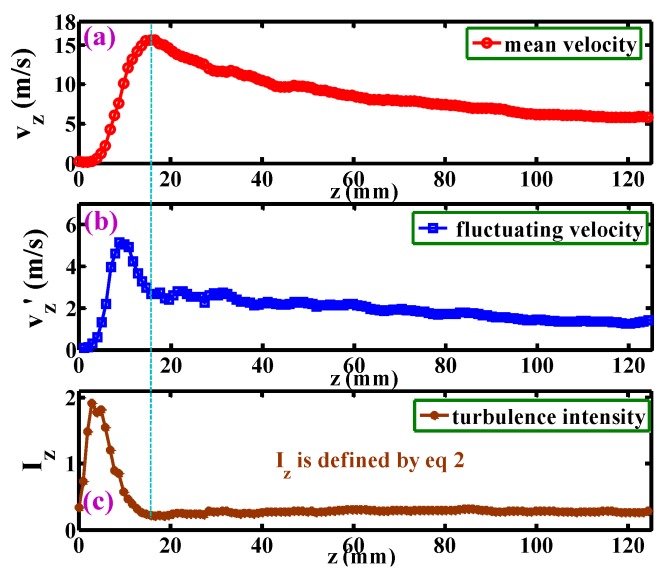
Development of (**a**) the mean velocity, *v*_z_, (**b**) the fluctuating velocity, *v*_z_’, and (**c**) the turbulence intensity, *I*_z_, along the centerline.

Figure 8a shows the development of the mean velocity, *v*_z_, along the centerline. The *v*_z_ rapidly increases to a peak of 16 m s^−2^ at *z* = 15 mm, and then gradually decreases to 6 m s^−2^ at about *z* = 120 mm. According to the Reynolds similarity criterion (i.e., Equation (1)), the maximum velocity of the “spinning die” melt-blown airflow will be at the position of *z* = 3 mm (the structure of the “spinning die” is 1/5 of the “model die”), which coincides with the previous CFD results [25]. Figure 8b shows the development of the fluctuating velocity, *v_z_*’, along the centerline (*v_z_*’ is defined as the average value of the difference between the instantaneous velocity and the mean velocity). The tendency of *v_z_*’ is similar to Figure 8a, however, the peak of *v_z_*’ = 5.5 m s^−2^ locates at *z* = 10 mm, indicating that the position of the maximum *v_z_*’ occurs earlier than the position of the maximum *v*_z_. Figure 8c shows the development of the turbulence intensity, *I_z_*, along the centerline. The *I_z_* is described as

(2) $$I_{z}=\frac{\delta_{z}}{v_{z}}=\frac{\sqrt{\frac{\sum_{1}^{N}{\left(v_{tz}-v_{z}\right)}^{2}}{N}}}{v_{z}}$$

where *v_tz_* is the air instantaneous velocity along the *z*-direction; *v_z_* is the mean velocity along the *z*-direction; *σ*_z_ is the standard deviation of *v_tz_*; *N* is the number of the *v_tz_* in the time segment of 10 s.

The peak of *I_z_* locates at about *z* = 5 mm, which is earlier than both positions of the maximum *v*_z_ and the maximum *v_z_*’; this result agrees with the CFD simulations [17]. It is expected that the polymeric fiber in this region undergoes prominently instable drag by the air, resulting in uneven fiber diameters. In addition, the *I_z_* decreases sharply to a minimum value at the position of *z* = 15 mm after the peak, and there is no obvious changing of *I_z_* in the region of *z* > 15 mm.

**Figure 9:**
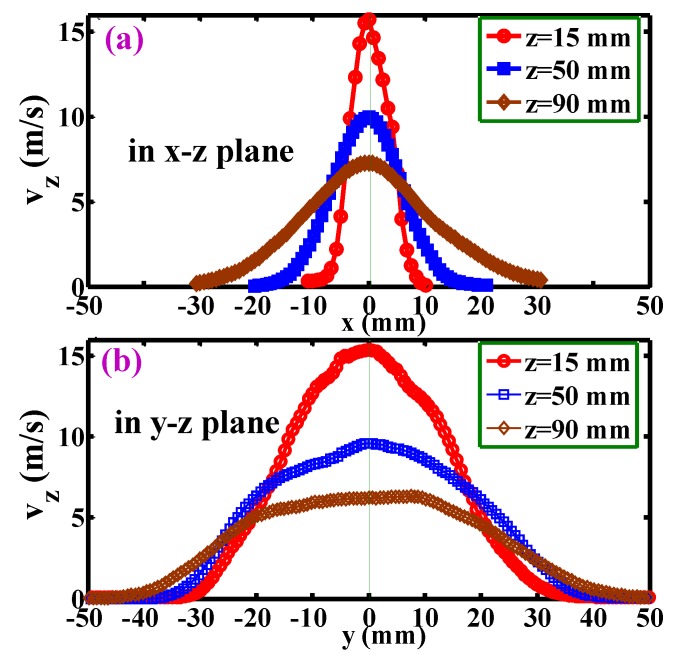
Development of the mean velocity, *v*_z_, for positions below the die along (**a**) the *x*-coordinate and (**b**) the *y*-coordinate at different *z*-levels. Positions for (**a**) and (**b**) are in the *x-z* plane and the *y-z* plane, respectively.

Figure 9 shows the development of the mean velocity component, *v*_z_, for positions below the die face. The profiles of *v*_z_ along the *x*-direction and *y*-direction are symmetrical about the ±*x*-coordinate and ±*y*-coordinate, respectively. In addition, they appear as a unimodal distribution. For Figure 9a, the maximum *v*_z_ at different *z*-levels decreases further away from the die face. The maximum velocity was about 16 m s^−2^ at the *z* = 15 mm level, and then decreased to about 7 m s^−2^ at the *z* = 90 mm level. However, the attenuation-rate of *v*_z_ along the *x*-coordinate gradually decreased for the positions far away from the die face. Figure 9b shows the development of the *v*_z_ along the *y*-direction below the die face, and in comparison with Figure 9a, the decay of the *v*_z_ is slower at the same *z*-levels. Although the length of the air slots was 30 mm along the *y*-direction, an expected 30 cm wide velocity distribution platform did not occur (still unimodal distribution). That means the length of the air-slot (i.e., 30 cm) is not long enough to allow the airflow to be a two-dimensional flow field.

**Figure 10:**
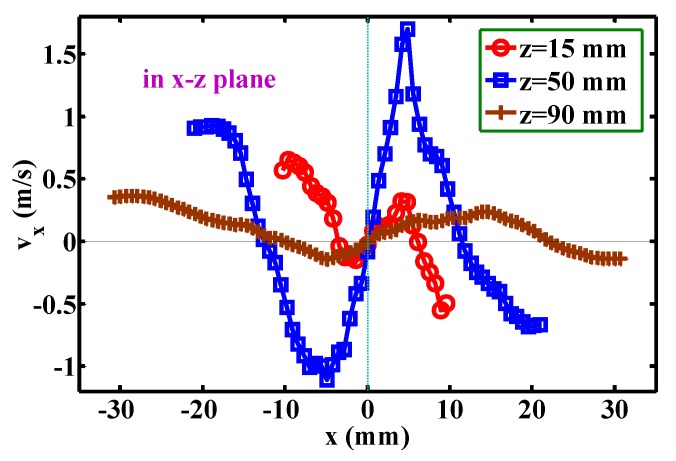
Development of the mean velocity component, *v*_x_, for positions below the die face. All positions are in the *x-z* plane.

**Figure 11:**
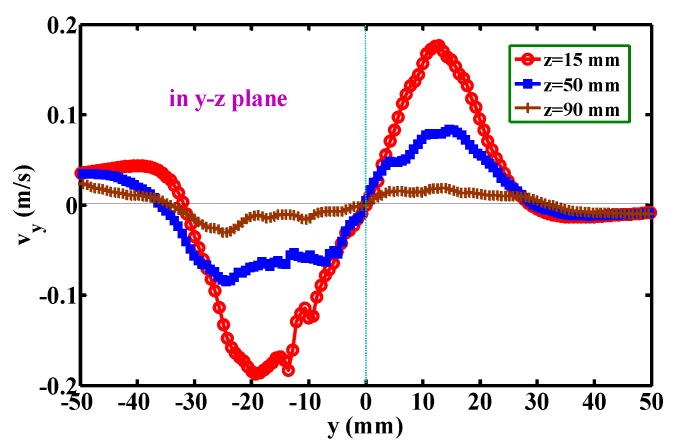
Development of the mean velocity component, *v*_y_, below the die face. All positions are in the *y-z* plane.

Figure 10 shows the development of the mean velocity component, *v*_x_, below the die face. The profiles of *v*_x_ along the *x*-coordinate are symmetrical about the point of *x* = 0, *v*_x_ = 0. Note that the positive *v*_x_ in the region of *x* > 0 and the negative *v*_x_ in the region of *x* < 0 represent the lateral diffusion of the airflow. The widths of the lateral diffusion are about 8 mm (i.e., from *x* = −3 mm to *x* = 5 mm), about 26 mm (i.e., from *x* = −13 mm to *x* = 13 mm), and about 32 mm (from *x* = −11 mm to *x* = 21 mm) for the levels of *z* = 15, 50, and 90 mm, respectively indicating that the width of lateral diffusion gradually increases further away from the die face, which corresponds to the lateral width of the airflow, as shown in Figure 4. Figure 11 shows the development of the mean velocity component, *v*_y_, below the die face. The profile of *v*_y_ along the *y*-direction is similar to Figure 10. However, the width of the lateral diffusion at different *z*-levels are almost constant, i.e., about 60 mm. Additionally, the maximum *v*_y_ is much less than the maximum *v*_x_ in Figure 10. This result indicates that the fiber undergoes smaller lateral air force along the *y*-direction than the air force along the *x*-direction. This phenomenon explains why the fiber whipping amplitude in the *x-z* plane is obviously larger than the amplitude in the *y-z* plane [16].

### 3.3. Evolution of Turbulent Airflow

The evolution of turbulent airflow is demonstrated by tracing the ejected process of the airflow. The evolutive process of turbulent airflow was carried out as follows. The airflow was firstly stopped. However, the PIV began to work, i.e., the laser emitted a pulsed laser, and the CCD camera correspondingly captured the vacant images. Then, the airflow with traced particles began to eject. The process from the air that had just been ejected, to the air stream becoming a normal airflow, was imaged by the CCD camera.

**Figure 12:**
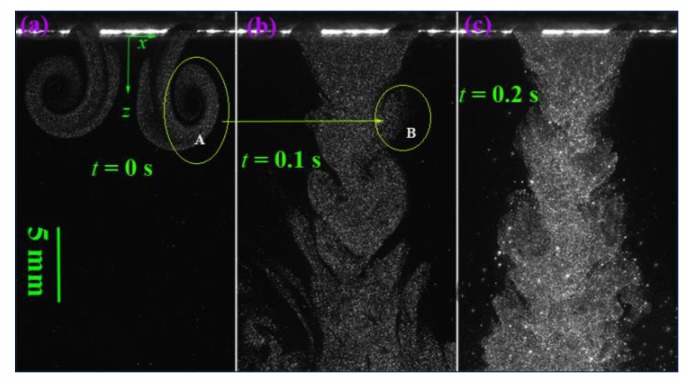
The evolution of air turbulence at continuous time instants of (**a**) *t* = 0 s, (**b**) *t* = 0.1 s, and (**c**) *t* = 0.2 s. The real size for each sub-image is 35.9 mm × 21.5 mm.

Figure 12 shows the evolution of airflow at continuous time instants. Figure 12a shows the structure of the air stream just ejected from the air slots at a time instant of *t* = 0 s. It shows that the two air streams evolve into reversed turbulent eddies after they were ejected from the air slots. Each eddy (as “A” shows) shows prominent rotating characteristics and the air entrainment effect still can be found. These two eddies were caused by not only the collision effect of the two ejected airstreams but also the resistance effect of stationary air to the airstreams. Figure 12b shows that the structure of turbulent airflow at a time instant of *t* = 0.1 s consists of large-scaled fishtail-like eddies (as “B” in Figure 12b), which evolved from the turbulent eddies shown in Figure 12a. As time went on, the fishtail-like eddies continued to deformation and became the S-shaped eddy shown in Figure 12c.

## 4. Conclusions

In the present work, the turbulent airflow in slot-die melt blowing was measured with the approach of PIV. The PIV results demonstrate the visualized structure of the turbulent airflow. It shows that the ejected two air streams from a pair of slots integrate immediately and begin to form an S-shaped turbulent structure. The PIV results also show that the position of the maximum turbulent intensity occurs earlier than the position of the maximum fluctuating velocity, which occurs earlier than the position of the maximum velocity. The evolution of turbulent airflow during melt blowing shows that after the two air streams eject from the slots, they firstly evolve into reversed turbulent eddies, then evolve into large-scaled fishtail-like eddies, and finally become the S-shaped eddy.

This work also explored some relationships between the turbulent airflow and the fiber whipping path. The fiber whipping path, which consists of two groups of arcs, appears to be forced by the S-shaped turbulent airflow, and the anisotropism of lateral expansion of the fiber whipping in the *x-z* plane and the *y-z* plane is due to the *v*_x_ along the *x*-direction being obviously larger than *v*_y_ along the *y*-direction. The present work on turbulent airflow provides a preliminary explanation for the specific fiber whipping motion in slot-die melt blowing.
